# So let me find my way, whatever it will cost me, rather than leaving myself in darkness: experiences of glaucoma in Nigeria

**DOI:** 10.3402/gha.v9.31886

**Published:** 2016-12-06

**Authors:** Fatima Kyari, Clare I. Chandler, Martha Martin, Clare E. Gilbert

**Affiliations:** 1 International Centre for Eye Health, Department of Clinical Research, London School of Hygiene and Tropical Medicine, London, United Kingdom; 2 Department of Ophthalmology, College of Health Sciences, University of Abuja, Abuja, Nigeria; 3 Department of Global Health and Development, Faculty of Public Health and Policy, London School of Hygiene and Tropical Medicine, London, United Kingdom; 4 Initiative for Community and Rural Eye Care, Kaduna, Nigeria

**Keywords:** glaucoma, blindness, vision loss, late diagnosis, early detection, care pathway, Nigeria

## Abstract

**Background:**

Blindness from glaucoma is associated with socio-economic deprivation, presumed to reflect poor access to care and poor adherence to treatment.

**Objectives:**

To determine why people with glaucoma are presenting late for treatment and to understand access to glaucoma care. Additionally, we sought to identify what patients and the community know, do and think about the condition and why the poor are the most affected with glaucoma blindness.

**Design:**

Study participants were from four communities and two hospitals in Abuja-FCT and Kaduna State, Nigeria. A total of 120 participants were involved, including 8 focus group discussions, 7 in-depth interviews with blind/visually impaired glaucoma patients, 5 rapid direct observation visits with these patients and 13 exit interviews of glaucoma patients in the hospital. The data were analysed using content analysis, interpreting participant experiences in terms of three key steps conceptualised as important in the care pathway: what it takes to know glaucoma, to reach a diagnosis and to access continued care.

**Results:**

This article presents multiple narratives of accessing and maintaining glaucoma care and how people manage and cope with the disease. People may be presenting late due to structural barriers, which include lack of knowledge and awareness about glaucoma and not finding an appropriately equipped health care facility. What keeps glaucoma patients within the care pathway are a good hospital experience; a support structure involving family, counselling and shared patients’ experiences; and an informed choice of treatment, as well as agency. The high cost of purchasing care is a major factor for patients dropping out of treatment.

**Conclusion:**

The findings suggest the need to address economic and social structural drivers as glaucoma presents another case study to demonstrate that poverty is a strong driver for blindness. There is also a need for clear glaucoma care pathways with early case finding in the community, two-way referral/feedback systems, well-equipped glaucoma care hospitals and better eye health care financing.

## Introduction

Glaucoma is the leading cause of avoidable irreversible blindness globally (1). In Nigeria, the recent national survey of blindness showed the prevalence of glaucoma to be high (5%) among adults aged 40 years and above, 94% of those with glaucoma were undiagnosed and untreated and one in five were blind (2). Poverty and socio-economic deprivation are significant risk factors for blindness from glaucoma (3–5). In a recent study of glaucoma patients in north-eastern Nigeria, 76% were already blind when they presented to the hospital with older age, poor knowledge of glaucoma, rural residence and living more than 10 km from the hospital being associated with blindness at presentation (6). Glaucoma blindness, therefore, reflects disparity in access to care. Additionally, there is a correlation between worsening quality of life and increasing severity of disease (7, 8).

Recent advances in technology for early diagnosis of glaucoma, greater therapeutic options and possibilities for treatment monitoring reduce the probability of blindness among patients in the care system in industrialised countries (9). Hence, blindness from glaucoma and the negative impact on quality of life are avoidable. The biomedical description of glaucoma is based on a known set of symptoms and signs including loss of sight, loss of visual field and raised intraocular pressure. Once the diagnosis has been made and the disease named, treatment is recommended to prevent further vision loss and maintain quality of life. Late presentation is when a person presents with biomedically severe/advanced disease in the worse-affected eye where visual acuity is <3/60, cup:disc ratio is >0.8 and central visual field is <10 degrees.

In this qualitative study, our main question was whyare people with glaucoma presenting late for treatment, with severe/advanced disease, rather than presenting with moderate disease at a point when progression to blindness can be slowed with biomedical intervention. We also sought to identify what patients and the community know, do and think about the condition and why the poor are the most affected with glaucoma blindness. We studied sociocultural contexts that impinge on the delivery of interventions for glaucoma. Providing a critical perspective on services for glaucoma would enable strategies to be developed to deliver more responsive and, hence, effective interventions and care, both for individuals and communities most affected in Nigeria and other sub-Saharan countries with similar high prevalence of glaucoma who also share similar socio-economic and socio-demographic characteristics.

## Methods

This study employed qualitative methods to assess participants’ knowledge and treatment of glaucoma using our clinical perspective as the benchmark.

### Conceptual framework

**Fig. 1:**
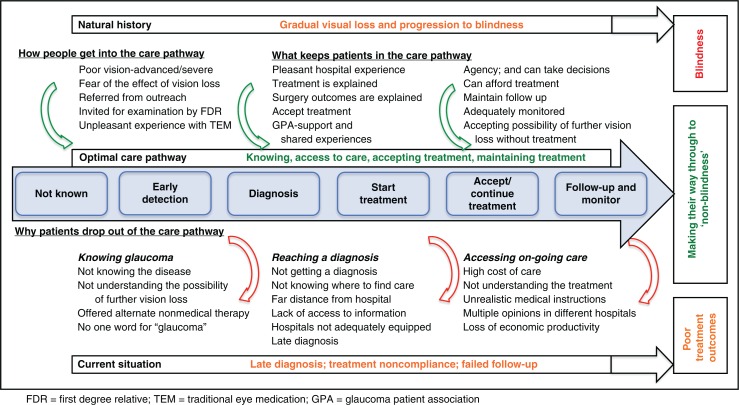
A conceptual framework for the glaucoma care pathway.

We conceptualised a framework for an optimal glaucoma care pathway (see the central flow in Fig. 1) and imagined that patients should take those steps to avoid blindness. The pathway involved getting to know glaucoma, having a diagnosis, accepting the treatment offered, compliance with treatment and maintaining monitoring and follow-up. In order to obtain data from multiple perspectives, the study employed a number of methods: focus group discussions (FGDs) held in the community, in-depth one-to-one interviews (IDIs) with blind/visually impaired glaucoma patients and their direct observation (DOs), and exit interviews (EIs) of glaucoma patients in the hospital. This range was selected in order to have a wide range of respondents at different sites so as to corroborate findings between people in the community and patients that have accessed care.

### Study area

The study areas were Abuja, Federal Capital territory, the capital city of Nigeria situated in the central part of the country; and Kaduna State in the north-west geo-political zone. Abuja comprises six local councils, two of which were included in our study: Bwari and Gwagwalada, in which we included one urban location (Kubwa) and one rural location (Sheda), respectively. In Kaduna, we included one urban location, Tudun Wada, and one rural location, Sabon Birni. Both areas have government and mission hospitals that provide eye care. We selected two hospitals that provide glaucoma services, one in each of the two areas. Hospital 1, located in Gwagwalada, Abuja, is mission-run, and Hospital 2, located in Kaduna, is government-owned.

### Participant selection and sample size

**Fig. 2:**
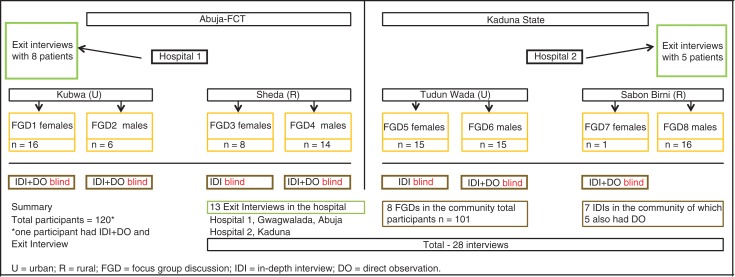
Sampling strategy and sample size for the patient and community perception study.

The study was based on eight FGDs held in the community, seven one-to-one IDIs with blind/visually impaired glaucoma patients, five of whom were directly observed in the community, and 13 EIs of glaucoma patients in the two selected hospitals (Fig. 2), consisting of a total of 120 participants. The fieldwork was conducted between January and March 2012.

The study team consisted of the researcher who is also the first author (FK) of this article, research assistant, also a co-author (MM), and the note-taker/field assistant. The two assistants were trained for data collection in health care research. Training of the assistants by the researcher included a discussion on the overview of the study aim and objectives, procedures, participant recruitment and interview/discussion techniques, and possible challenges and how to overcome them.

Purposive sampling was used to select hospitals and participants. Community-based research facilitators (HR, CO, FE and ES) were involved in selecting the four communities outlined above. The research assistant together with the community-based facilitators, who were involved in community-based rehabilitation of patients with disability or had been on outreach, identified and recruited the participants in the community for the FGDs and the IDIs (and DOs). There were two local ophthalmologists (FA and TN) who facilitated the selection of participants for the EIs in their hospitals. No incentives were provided to participate, but all were offered free eye examination and referral where necessary and refreshments were provided.

Discussions and interviews were conducted in English, Hausa or Pidgin English by FK or MM accompanied by the note-taker, with little need for an interpreter as both interviewers were multilingual in the languages of discussion. However, one FGD and one EI were conducted in Gbagyi where a translator was required. All interviews/discussions were recorded with a digital recording device, and notes were taken.

### Focus group discussions

Two FGDs with members of the community were held in each of the four communities, that is, total of eight FGDs, conducting separate discussions for female and male groups in order to have a relaxed atmosphere and foster openness. Participants were aged 30 years and above and included a community leader, visually impaired/blind, and normal-sighted community members. The FGDs were held in a convenient private meeting area within the community. Written informed consent was obtained from participants after explanation of what would take place and their basic demographic data were recorded.

A topic guide was followed in order to stimulate discussion and bring out potential factors exploring the knowledge and practices in relation to eye diseases and blindness in general, and glaucoma in particular, their perception of risks and concept and understanding of blindness. We also explored their health-seeking processes.

After each FGD, the study team reviewed the audio recording, and challenges and need to include more probing questions were discussed.

### In-depth interviews

IDIs were conducted with glaucoma patients in the community who were visually impaired or blind and had not accessed treatment or had had treatment whether successful or not. The IDIs were conducted in the participant’s home in a place which provided privacy. We carried out IDIs using a narrative approach: ‘tell me about your eye problem/disease …’ and with a topic guide for prompt questions in order to explore participants’ knowledge about their disease, what symptoms triggered them to seek care, difficulties in seeking care, their perception of glaucoma as a cause of blindness and the cost of finding care.

### Direct observation

DO involved shadowing the participants to observe how their everyday lives were affected, particularly with regard to their home environment and interactions within the community. We selected IDI participants who gave us the opportunity to observe them in their homes. We had a checklist of observations, which included how they interacted with their family members and how members of the community approached and related with them. We also observed how independent they were in terms of mobility, use of everyday gadgets such as mobile phones and telling the time.

### Exit interviews

One-to-one EIs were conducted, and participants were asked to narrate their experience of the hospital visit and what they felt about the diagnosis and treatment. This also included their positive experiences, rather than only barriers to accessing health care. They were also asked about triggers that led them to seek care, whether their condition was explained to them and what they understood about glaucoma and the effects of their sight loss on their everyday lives, and how much money they had spent on eye care. They were also asked about their knowledge and use of traditional (non-medical) eye medication (TEM) and what they would tell new patients who had been diagnosed with glaucoma. Participants ranged in age from 29 to 74 years.

## Data handling and analysis

The audio recordings were transcribed in the language of discussion. Hausa transcripts were translated into English. English translations were crosschecked and finalised by FK. English transcripts of FGDs, IDIs (and DOs) and EIs were imported into NVivo 10 (QSR International Pty Ltd., Victoria, Australia). The data were accessible to only the researcher and co-authors.

### Data immersion

The researcher became familiar with the data through conducting, transcribing and translating most of the interviews. Transcripts were read carefully and coded line by line.

The data were analysed using content analysis, interpreting participant experiences in terms of three key steps conceptualised as important in the care pathway: ‘knowing glaucoma’ which gave a perspective of people’s experience and not compared to what they should know; ‘reaching a diagnosis’ which stems from knowing glaucoma and as a prerequisite for treatment; and ‘accessing on-going glaucoma care’ which includes issues of cost of care, decisions on treatment and non-medical alternatives that people might be offered. Within each step, we identified explanatory themes. The themes were developed based on initial reading through the transcripts. A coding template was set up for the three themes that emerged from the data and agreement of categorisation reached through discussion and review by the co-authors (CC and CG). We remained open to additional codes and new themes that emerged during analysis. Additional codes were applied in order to identify what it really meant to people to have glaucoma and how debilitating it was. Initial coding was done by hand, and subsequent categorisation and archiving were done using NVivo 10 (QSR International).

### Ethics

Ethical approval was obtained from the Ethics Committee of the London School of Hygiene and Tropical Medicine, UK, and the Nigeria National Health Research and Ethics Committee. Written informed consent was obtained from the participants. We specifically asked to record interviews, take photos and use anonymous quotes. Confidentiality and anonymity were maintained. The study did not interfere with any treatment that patients were receiving. Participants in need of further management for their eye condition were attended to and referred to the appropriate facility if necessary.

## Results

### Study participants

All participants in the FGDs (*n*=101) were aged 30 years and above (Table 1). Basic demographic characteristics of the IDI and EI participants (*n*=19) are shown in Table 2.

**Table 1:** Demographic characteristics of participants of focus group discussions in the community

	Number of participants (%)		
	
	Rural (Sheda and Sabon-Birni)	Urban (Kubwa and Tudun-Wada)	Total
Gender			
Female	19 (39)	31 (60)	50 (50)
Male	30 (61)	21 (40)	51 (50)
Age (years)			
30–45	6 (12)	11 (21)	17 (17)
46–60	17 (35)	17 (33)	34 (34)
61 and older	4 (8)	22 (42)	26 (26)
Not indicated	22 (45)	2 (4)	24 (24)
Occupation (current/retired)			
Civil service officer	–	6 (12)	6 (6)
Driver	–	2 (4)	2 (2)
Farmer	9 (18)	1 (2)	10 (10)
Housewife	6 (12)	16 (31)	22 (22)
Military	–	1 (2)	1 (1)
Office assistant/cleaner	1 (2)	3 (6)	4 (4)
Student	–	1 (2)	1 (1)
Teacher/lecturer	2 (4)	3 (6)	5 (5)
Trader/business	3 (6)	16 (31)	19 (19)
Not indicated	28 (57)	3 (6)	31 (31)
Total participants	49 (100)	52 (100)	101 (100)
Language of discussion			
English	–	2 (50)	2 (25)
Gbagyi^a^	1 (25)	–	1 (12.5)
Hausa	3 (75)	2 (50)	5 (62.5)
Total FGDs	4 (100)	4 (100)	8 (100)

FGD, focus group discussion.

a An interpreter was used in this FGD.

**Table 2:** Basic demographic information for the participants who had in-depth interviews in the community and exit interviews in the two selected hospitals

No.	Code	Gender	Age^a^ (years)	Occupation	Available clinical description
1	IDI-1; EI-2	F	62	Housewife (military)	VA: RE 6/9, LE HM; BE CDR 0.9
2	IDI-2	M	60	Retired as military nurse	VA: NPL BE; BE CDR 1.0
3	IDI-3	M	59	Stopped driving	VA: NPL BE
4	IDI-4	M	45	Trader	VA: RE PL, LE NPL
5	IDI-5	M	75	Butcher	VA: RE CF, LE 6/9; RE CDR 1.0, LE CDR 0.9; LE trabeculectomy 12 years
6	IDI-6	F	67	Housewife	BE not seeing. Sees some shadows
7	IDI-7	M	43	Teacher	VA: RE NPL, LE NPL; Diagnosed glaucoma and had RE trabeculectomy 9 years ago; then had RE vitrectomy for endophthalmitis 6 years later
8	EI-1	M	29	Works with a trading company	RE not seeing
9	EI-3	M	56	Stopped work	RE cloudy; LE not seeing
10	EI-4	M	52	Senior civil servant (Intelligence department)	Diagnosed glaucoma 8 years ago; Had triple procedure in first eye and trabeculectomy only in second eye
11	EI-5	M	40	Farmer	LE not seeing
12	EI-6	M	74	Lecturer at college of education	One eye blind since early adulthood. Had cataract surgery and diagnosed glaucoma in the only eye
13	EI-7	M	58	Electrician, works with contractor firm	One is bad. Diagnosed glaucoma more than 5 years ago
14	EI-8	M	60	Farmer	RE not seeing clearly; had RE trabeculectomy
15	EI-9	F	53	Theatre nurse	RE worse; had RE trabeculectomy
16	EI-10	M	53	Worked in telecommunications. Made redundant due to company closure	BE seeing ok
17	EI-11	M	42	Vehicle insurance officer (civil servant)	LE not seeing – had surgery in LE prior to diagnosis of glaucoma in BE
18	EI-12	M	70	Dependent on children	RE not seeing
19	EI-13	M	62	Mechanic, contractor	One sees well, other not much

IDI, in-depth interview; EI, exit interview; VA, visual acuity; RE, right eye; LE, left eye; HM, hand motions; BE, both eyes; CDR, cup-to-disc ratio; NPL, no perception of light; PL, perception of light; CF, counting fingers.

a Some ages were estimates.

The findings represent narratives of accessing and maintaining glaucoma care and how people managed and coped with the disease. It is important to know that from many people’s perspective, there is no care pathway; it is just life, the lived reality. Thus, other aspects we explored were the coping mechanisms of patients with glaucoma and the consequences of fear of the effect of sight loss, feelings of isolation, abandonment, stigmatisation and loss of autonomy as well as financial stress and loss of economic/social productivity. The coping mechanisms were within sociocultural constructs of faith in God and support from family, friends and community. It was not only about coping with the disease, but also about coping with the social situation they were in.

Some quotes have been paraphrased to ease reading without losing the context and meaning captured in the discussion. Furthermore, four cases are presented to illustrate the different themes.

### Knowing glaucoma

Blindness is generally considered a serious problem. However, participants avoided use of the term ‘blindness’ or ‘*makanta*’ (Hausa), rather they would say ‘eye problem’ or ‘*matsalar ido’* (Hausa). Participants’ description of blindness often indicated a total loss of vision, attaching a morbid reality to it, while those with poor vision did not always define themselves as blind.

There was generally poor knowledge of eye diseases as understood in biomedicine and lack of access to information. Most participants got information about health issues from the radio. Other sources are places of worship (church and mosque), through reading and interaction with neighbours or health workers. They, however, would rely on information given them by the doctor or at the hospital during health talks. They described symptoms without giving specific names for eye disease except for cataract (*yana*) and corneal opacity (*hakiya*), though sometimes they interchanged description of the two. Most participants had not heard about glaucoma, and they recognised that self-medication with inappropriate medicines would cause delay in seeking treatment.

None of the patients had heard of the term ‘glaucoma’ before their diagnosis. They did not bundle their symptoms and experiences as a disease entity, and there was no reference to any biomedical category. Some of their symptoms such as redness and tearing were often considered to be common and less sight-threatening eye conditions. Different things happened over time, such that by the time they sought treatment, there was severe vision loss in at least one eye. Participant EI-8 said ‘I cannot see very well. Like I am in the dark’, and EI-4 felt indeed glaucoma is a *silent thief of sight*.

People’s experiences differed, and some participants came to know things were not quite right with their vision, some of which may indicate visual field loss. For example, not being able to see the right underarm while shaving was illuminating for EI-1, whereas EI-13 could only see clearly through the corner of the eye, and IDI-7 could not see people’s body completely.

Factors related to *not* knowing glaucoma included stigma around blindness and poor vision so that they would not talk about it; misconceptions on causations of eye diseases; and general lack of awareness, knowledge and access to information about glaucoma and eye diseases. These factors led many participants in the care system to present with late disease and to access multiple opinions in different hospitals, *hopping and hoping*.

### Reaching a diagnosis

Knowing glaucoma is part of reaching a diagnosis. Even when they got to know they have glaucoma, some have no information on what to do about it. In health care centres where there are no trained health-workers or appropriate equipment, then one cannot make a diagnosis of glaucoma. Participants did not have a designated entry point into clinical care, and there was no system of referral. Participants did not know where to find care or what treatment to expect nor did they appreciate the possibility of future sight loss without treatment, which led some to visit multiple providers – going from one health facility to another. As IDI-5 experienced: ‘I kept going (to the hospital). Then, the hospital was moved to Shika. Then I started going to Eye centre. Thereafter I went to Dan Tsoho. I was n’t satisfied, so I went to Zaria. Then I was told that there was a hospital in Kano. I started going there. I was not comfortable so I changed to another hospital, right there at Kano’.

The high cost of finding care contributed to these difficulties, but EI-11 had agency and was determined to find and maintain care (Box 1). On the contrary, inability to make autonomous decisions for one’s own benefit contributed to delays in making a diagnosis (see Box 2: IDI-2). Some participants mentioned distance to hospital was a reason for their poor access to care and felt they had no bargaining power and could not request for services to be brought closer to them.

***Box 1*. EI-11 illustrates late presentation, getting to know glaucoma, agency, accepting the possibility of further vision loss without treatment and maintaining continued care**

EI-11 is a 42-year-old senior civil servant:

When I discovered that I had eye problem … one eye was seeing, one eye was not seeing, I said I cannot continue like this, let me find my way to FMC but I was stopped by a friend who recommended a private clinic. I did not know it was run by a nurse. There, I was told I had glaucoma. He did not give much guidelines and explanation. Had I got guidelines and explanation, it would have not reached up to this stage at which I am in now. Later, after one year plus, I changed to a private doctor. He also said I had glaucoma and one of my eyes was severely affected. That is the left eye. Then he explained glaucoma. And ah, that’s how I started to know about glaucoma.

Because at the private hospital, … you will spend much and much and much and much. I asked him to give me a referral letter to NEC. He said ‘why refer if it’s what I can do?’ Though he’s a qualified doctor, he’s a doctor. Then I later thought it over … I said kai … (sigh) I’m educated so let me find my way. Whatever it will cost me, let it cost me rather than leaving myself in darkness. And I don’t want to be in the dark!

Here, they explained ALL (his emphasis) things to me. And they said ANY (!) nerves, or ANY (!) eye sight that glaucoma destroys, it is destroyed for life. So that’s why I said I cannot stay and continue looking at it … then leaving myself in darkness. Because I am still young, I don’t know how long I will live in the world and my eye … Then that I’m finished. So that’s why I normally maintain the period I’m given for appointment. I don’t fail it. I don’t fail it. Yes.

***Box 2*. IDI-2 illustrates lack of autonomy to take decisions, not understanding the treatment and feeling of abandonment but accepting the situation he is in**

IDI-2 retired as a staff sergeant after 35 years in the military as a nurse. He is blind in both eyes from glaucoma. He is a widower and lives with 3 of his 5 children, the youngest being 11 years old. The oldest son is away on military service, and the oldest daughter is at University. Interviewing him was my second IDI of a blind person in the community.

‘When I was diagnosed with glaucoma in 2004’, the doctors suggested surgery. However, my preparation for retirement from the military stopped the discussion of surgery. Then things happened so quickly – I was retired, had to leave the barracks official accommodation to my uncompleted house which was yet to be roofed. ‘At the time I moved to this place I could see and move around everywhere’. That was 2007.

‘At the hospital, I had been receiving treatment but there was no improvement. I went to another hospital’. I continued treatment until I got fed up … ‘Anywhere I went, they would say timolol, timolol … ’

When asked about how he copes being blind – ‘It is not easy … The children would just go away. Not that we don’t have … I have television, DVD, radio, anything that can make them happy to stay here. I don’t shout at them – there’s food, everything. I don’t know why … I’m not having peace of mind again. As I cannot see anything at all how can I go out myself? There is nothing I can do … The challenge is too much but there is nothing I can do. What can I do? Do I cry? If I cry, am I the first person to go blind? So there is nothing I can do than to accept it like that. So I have to thank God very well. ‘It is said in the Bible that in any situation you see yourself, accept it’.

Thus, the contributory factors for late diagnosis include poor knowledge about the disease, not finding an appropriately equipped hospital and inability to afford care.

### Accessing and maintaining glaucoma care

Hospital experiences varied considerably. However, a good hospital experience and obtaining appropriate information made a difference in patients’ understanding of their disease and gave them hope.

Family members are cardinal in decision-making for choice of treatment options, and participants would often discuss with them before taking decisions. Thus, patients and their carers/family need to fully understand the disease and the implications of choices of treatment. Once a diagnosis of glaucoma is made and choice of treatment is considered, physicians need to discuss treatment options with the patient and family. This is also helpful for identifying first-degree relatives with glaucoma. IDI-4’s older brother was already blind at the time of diagnosis. IDI-4 also had late diagnosis and could not sustain medical treatment, and he gradually became blind. His younger brother was also diagnosed late but had surgery in the only seeing eye and this helped to maintain his vision.

Some participants had unpleasant experience with TEM (see Box 3: IDI-6).

***Box 3*. IDI-6 illustrates lack of access to medical care, use of traditional eye medication and not understanding treatment**

IDI-6 is a 67-year-old housewife. Her husband is the District Head. She is blind in both eyes from glaucoma. She has never been to a hospital/clinic nor been on biomedical treatment:

‘My eyes kept hurting and hurting and then they brought me some perfume which I sprayed on the eyes. But they got worse. Then they said I should take a frog and rub it on the eyes. I said I couldn’t do that. Then my husband picked up the frog and rubbed it on my eyes and when he threw the frog, it died. The eyes got better, there was no pain again. Then they gave me kohl which I kept applying for months and years and the vision continued dimming and getting worse. Now, that is my story’.

Asked why she didn’t go to the hospital –

‘I have not been to hospital. The first time they came (on outreach), I was told I needed operation. But some people said to my husband that if I went, they would sever my eye nerves (*za’a tsinke jijiya*) so I refused to go since then’.

Hospital charges and cost of medicines were a great concern, and in some cases, these contributed to poor compliance with medical therapy. IDI-4 could not keep up with buying medicines due to cost, and IDI-7 lamented that all he had spent was to no avail. Inability to afford hospital costs precluded patients from getting and maintaining treatment. FGD/4/P6 mentioned ‘Actually, in the hospital, they asked me to pay about N60,000 (£240). But with that amount of money requested, I just put the paper in my pocket and went back home. One who has not even N100 (£0.40p) at home, they ask for N60,000 (£240); how can you even begin to get that?’ On the contrary, EI-4 alluded to the availability of health insurance as being beneficial for enabling access.

In terms of getting information about their disease, some perceived a hierarchical doctor–patient relationship characterised by one-way communication, with the patient not having courage to ask for explanations. Some participants felt this was because clinicians have enormous social responsibilities despite their busy work schedule and much is expected from them. Rather, they were satisfied with a one-to-one guidance and counselling on their disease.

Having a forum such as a glaucoma patient association would promote interaction between patients, with representation for actively addressing challenges in accessing care and treatment and obtaining social support. Participants believed that shared experiences would enhance ability to make informed choices and staying in treatment.

What keeps glaucoma patients within the care pathway are a good hospital experience; a support structure involving family, counselling and shared patients’ experiences; and an informed choice of treatment, as well as agency – knowing about glaucoma and being able to do something about it. The cost of treatment is a major factor for patients dropping out of treatment.

### Having glaucoma and coping mechanisms

IDI-2 expressed feelings of isolation and abandonment and loss of value to his children and friends (see Box 2), while some participants note that their visual impairment should not define who they are. IDI-5 felt awful for being called ‘blind-man’ (*makaho*). Likewise, IDI-7 who had been active in the community for about 40 years disliked being addressed as ‘blind-man’ (*makaho*): ‘why would people address me as such and alienate me?’

A diagnosis of glaucoma triggered anxiety: EI-1 said, ‘I had a breakdown. A shock went through my spine’; or perhaps regarded as a fate worse than death because ‘some people prefer to die instead of living with blindness’ (EI-4). There was also an emotional component as IDI-1 kept sobbing during the interview while saying ‘God, you know better, you will make it better’. Participants who had lost vision expressed their dismay in their inability to do certain tasks especially driving, writing and keeping their jobs. Some also had feelings of being a burden on those who assist them in their everyday activities.

Within the sociocultural framework of faith in God, some glaucoma participants did not see themselves as being blind and now suffering. Rather, they found ways to manage the situation. FGD/4/P3 said ‘I put my trust in God’ (see also Box 4: IDI-7). IDI-4 remains an important member of his community as the *Imam* who leads the congregation prayers in the local mosque. He finds strength in faith and accepts that everything in life would be left behind anyway.

***Box 4*. IDI-7 illustrates late diagnosis, difficulties in maintaining care, poorly equipped tertiary hospital, agency and coping mechanisms**

IDI-7 is a 43-year-old man, civil servant.

‘In 2003, the doctor said my left eye had end-stage glaucoma. I never knew that it was not seeing before I went to the hospital. It was when they tested me that I knew. They recommended surgery for the right eye. I had the first surgery in 2003 and continued to see without any problem. I would go for check-up regularly. Four years later in 2007, my seeing right eye got reddish. I got worried and went to see the ophthalmic nurse who recommended an eye drop. It was not available in my town so I bought it about 30 miles away. I saw my vision diminish gradually … The following day I went to NEC. I needed vitrectomy but they didn’t have the materials. Through a cumbersome process of referral, appointment and solicitation of funds, I had vitrectomy in Cairo, Egypt, two weeks later’.

‘My work keeps me busy. Currently I am heading a centre that teaches secondary school students English and Mathematics. We recruited 12 lecturers and we have about 6 classes with over 65 students’.

However, he expressed disturbing limitations as a public speaker and teacher. ‘You know when I address people, the only response I can hear from them is laughter or their voices, but I cannot see their eyes … That is one of my problems. Some people do not talk, but you can read them from their faces. But I cannot read those because I cannot see. It is only when somebody talks that I begin to know his feelings about me, so that is one of the disturbing things’.

‘It has stopped me from furthering my studies, Masters. After the first surgery, I could not read … But most importantly, I was not sacked from my job – that is a happy thing. I earn my salary and maintain my family’.

On his relationship with his family and community: ‘My family and friends have been very, very supportive. Especially my wife … The community too. If people could remove this sickness from me, the number of people that trooped into this house when I came back from hospital, they would have removed the sickness from me, on sympathy basis, I tell you … I gave everything to God’.

## Discussion

This study found that most people do not know about glaucoma, they are not aware when they have it, they do not know where to find care and they are faced with challenges in accessing and maintaining treatment because of poor infrastructure and high cost of care. A major trigger of seeking care was advanced loss of sight resulting in late diagnosis. Indeed, a person with glaucoma may frequently be unaware of the gradual loss of sight (10, 11). Loss of sight was often not discussed, and participants did not use the word ‘blind’ (*‘makanta’* in Hausa) to describe themselves. This silence can be seen to have allowed glaucoma to thrive without being diagnosed. As in Ghana (12), there was no specific name for glaucoma in the communities we studied. Similarly, the knowledge of glaucoma was low, as documented in previous hospital-based (13–16) and population-based (17–20) studies. Even in some developed economies, knowledge of glaucoma varies (21, 22). The lack of knowledge might have contributed to difficulty in appreciating the possibility of future sight loss if left untreated even though patients would live with future uncertainties (23). However, it was not only the silent nature of loss of sight due to glaucoma that precluded participants from finding or securing early care but also additional factors such as not knowing where to find care and not being able to afford or sustain care. In a study, where care is available and accessible, every patient followed up in a population-based survey had sought eye care (24), but the understanding of glaucoma was limited (25). In our study, those who had more agency, that is, resources and ability to take autonomous decisions, appeared to have found ways to access care.

In line with the United Nations resolution on Universal Health Coverage (26), a Global Action Plan (GAP) was developed for eye care (27). GAP aims to ensure that the diseases that cause blindness and visual impairment are addressed through universal standards of eye care, tailored according to local contexts and benefits of modern medicine. The GAP, inherently linked with vision 2020 ‘The Right to Sight’ (28), recognises the need to address problems of unequal access to eye care and to support weaker nations/communities to achieve those standards. This study provides information that will be useful to developing strategies for locally relevant eye care tailored towards optimal care.

In interpreting our findings, we identified the concept of structural violence as a useful way to understand and explain what could be causing people to be in the situation of lack of knowledge, late presentation and drop out from continued care. Structural violence originates from the perspectives that there is a disease and that the disease is disabling – for example, HIV/AIDS and there are structures that make the disease worse in others and structural inequalities that prevent access to care (29). When there are constraints and inequalities in socio-economic status and health systems structures, as we note here, that preclude avoidable blindness from being avoided, then there is structural violence (30). Put more succinctly, ‘structural violence is one way of describing social arrangements that put individuals and populations in harm’s way. The arrangements are structural because they are embedded in the political and economic organization of our social world; they are violent because they cause injury to people (typically, not to those responsible for perpetuating such inequalities) … neither culture nor pure individual will is at fault; rather, historically given, and often economically driven processes and forces conspire to constrain individual agency. Structural violence is visited upon all those whose social status denies them access to the fruits of scientific and social progress’ – Paul Farmer (29, 31). The concept of structural violence encourages us to reorient ourselves towards finding solutions, to critically engage the realities and recognise the situation due to structural inequalities and structural barriers, which cause harm, rather than passively accepting these as systemic inequalities (32). These structures of inequalities are invisible and embedded within the same political and economic systems such that no one individual or institution can be held accountable (31). For example, if a person goes irreversibly blind from glaucoma, which is avoidable, one might ask, who do we hold culpable?

In terms of agency, autonomy is related, partially, to having the ability or the resources to act freely. From the economic aspect, it could mean those who have a voice – for example, EI-11: ‘this is what I want’, wherein the socio-economic structure enables him. However, when people are unable to demand, the only agency they may have is to lament and leave – for example, IDI-2: ‘what else can I do?’ For those who had relatively better agency, for example, EI-11, they were able to seek care and navigate the difficult care pathway ‘rather than remain in the dark’. That was an active response. A somewhat passive response is accepting the situation and not taking a decision to go for a biomedical or traditional medicine but manage the ‘misfortune’ (e.g. IDI-6) and readjusting their social and family interactions (33, 34). This may not necessarily be interpreted as social suffering in the way people manage adverse situations, but takes into consideration coping mechanisms. The way they cope and the way they accept their situation might be because of the absence of care or structures that mean they cannot access care and they do not feel or know that getting better care is their right. It appears that health choices have been left to ordinary people to continue their own therapies, be it traditional medicine or self-medication from patent medicine stores or markets. This has been described as ‘subsistence’ health – where people are left to seek their own care (35), and traditional medicine is often sought where there were no alternative sources of treatment (36). In glaucoma, there is no system, no diagnostic category and no way of well-established management of the disease within traditional medicine. In fact, the more established practice of couching, which is the traditional manual manipulation for cataract, is widely practiced in Nigeria with very poor visual outcomes (37).

Furthermore, the narratives imply that whether one goes to the hospital or gets treated was a matter of fate and destiny, depending on the will of God. In a way, this submission to the will of God breeds acceptance of the situation. Some believe that loss of their sight is a test of their faith and perhaps an expiation of sins for a better life after death. Of note, however, is that coping may be a response to the absence of care or the structures that mean they cannot access care. This makes the coping mechanisms dynamic – people have resources and ability to manage the situation but they would not turn down the opportunity to have better care that is well explained, accessible and affordable.

### Limitations

There are limitations of this study. The analysis was undertaken using the transcripts of translation to English for three-quarters of the discussion. As such, some distinct expressions might have been lost in translation. Another limitation is that we conceptualised a care pathway and saw people who are not accessing or who are falling out of our imagined pathway. But from their perspective, there is no care pathway; for them, it is just life, embodied as lived realities. Additionally, a limitation of the structural violence perspective is that it labels one with a defining feature, for example, the glaucoma blind, whereas these patients did not see themselves as such.

### Recommendations

This population-based study provides a baseline and deeper understanding of access to glaucoma care. However, we recommend conducting a similar study in different settings for local content. A further recommendation is that in addition to offering biomedical/clinical service, providers need to collaborate and communicate effectively with patients, family members and carers so that they understand the disease, manage their expectations and be effectively supported to gain insight into the disabling consequences of blindness. Other needs are better eye healthcare financing, visual rehabilitation and social adaptations for people with visual impairment/blindness. A social policy and disability benefits would also ease some of the social suffering of blindness.

## Conclusion

In Nigeria, the reasons for late presentation imply the need for improving services for glaucoma. Availability and affordability of treatment need to be addressed so that hospitals are well equipped to manage glaucoma, incorporating early case-detection strategies with clear glaucoma care pathways and two-way referral/feedback systems.

## Paper context

Blindness from glaucoma is associated with late presentation and poor compliance to treatment. This study indicates that late diagnosis may be explained by structural barriers to accessing care: socio-economic deprivation, poor understanding of the disease and high cost of care. The findings suggest the need to address socio-economic structural drivers as glaucoma experiences demonstrate that poverty is a strong driver for blindness. There is also a need for clear glaucoma care pathways and better eye healthcare financing.

## References

[1] Pascolini D, Mariotti SP (2012). Global estimates of visual impairment: 2010. Br J Ophthalmol.

[2] Kyari F, Entekume G, Rabiu M, Spry P, Wormald R, Nolan W (2015). A population-based survey of the prevalence and types of glaucoma in Nigeria: results from the Nigeria national blindness and visual impairment survey. BMC Ophthalmol.

[3] Kyari F, Wormald R, Murthy GVS, Evans JR, Gilbert CE, Nigeria National Blindness and Visual Impairment Study Group (2016). Ethnicity and deprivation are associated with blindness among adults with primary glaucoma in Nigeria: results from the Nigeria national blindness and visual impairment survey. J Glaucoma.

[4] Cook C (2013). Socioeconomic status as a risk factor for late presentation of glaucoma in Canada. Can J Ophthalmol.

[5] Ng WS, Agarwal PK, Sidiki S, McKay L, Townend J, Azuara-Blanco A (2010). The effect of socio-economic deprivation on severity of glaucoma at presentation. Br J Ophthalmol.

[6] Abdull MM, Gilbert CC, Evans J (2015). Primary open angle glaucoma in northern Nigeria: stage at presentation and acceptance of treatment. BMC Ophthalmol.

[7] Onakoya AO, Mbadugha CA, Aribaba OT, Ibidapo OO (2012). Quality of life of primary open angle glaucoma patients in Lagos, Nigeria: clinical and sociodemographic correlates. J Glaucoma.

[8] Gothwal VK, Bagga DK, Rao HL, Bharani S, Sumalini R, Garudadri CS (2014). Is utility-based quality of life in adults affected by glaucoma?. Invest Ophthalmol Vis Sci.

[9] Malihi M, Moura Filho ER, Hodge DO, Sit AJ (2014). Long-term trends in glaucoma-related blindness in Olmsted County, Minnesota. Ophthalmology.

[10] Crabb DP, Smith ND, Glen FC, Burton R, Garway-Heath DF (2013). How does glaucoma look?: patient perception of visual field loss. Ophthalmology.

[11] Green J, Siddall H, Murdoch I (2002). Learning to live with glaucoma: a qualitative study of diagnosis and the impact of sight loss. Soc Sci Med.

[12] Opoku K, Murdoch IE (2013). Bridging the language barrier in health awareness. JAMA Ophthalmol.

[13] Bodunde OT, Daneil OJ, Onobolu OO, Ajibode HA, Awodein OG, Jagun OO (2006). Knowledge, attitude and health beliefs of glaucoma patients in a Nigerian hospital. Nig Med Pract.

[14] Eldaly M, Hunter M, Khafagy M (2007). The socioeconomic impact among Egyptian glaucoma patients. Br J Ophthalmol.

[15] Mbadugha CA, Onakoya AO (2014). The awareness, perceptions and experiences of primary open angle glaucoma patients in Lagos Nigeria. Sci Rep.

[16] Abdull MM, Chandler C, Gilbert C (2016). Glaucoma, ‘the silent thief of sight’: patients’ perspectives and health seeking behaviour in Bauchi, northern Nigeria. BMC Ophthalmol.

[17] Ntim-Amponsah CT, Winifried MKA, Ofosu-Amah S (2004). Awareness and knowledge of glaucoma and other diseases associated with blindness in a Ghanaian community. Nig J Ophthalmol.

[18] Balo PK, Serouis G, Banla M, Agla K, Djagnikpo PA, Gue KB (2004). Knowledge, attitudes and practices regarding glaucoma in the urban and suburban population of Lome (Togo)]. Sante.

[19] Yan X, Liu T, Gruber L, He M, Congdon N (2012). Attitudes of physicians, patients, and village health workers toward glaucoma and diabetic retinopathy in rural China: a focus group study. Arch Ophthalmol.

[20] Sathyamangalam RV, Paul PG, George R, Baskaran M, Hemamalini A, Madan RV (2009). Determinants of glaucoma awareness and knowledge in urban Chennai. Indian J Ophthalmol.

[21] Mansouri K, Orgul S, Meier-Gibbons F, Mermoud A (2006). Awareness about glaucoma and related eye health attitudes in Switzerland: a survey of the general public. Ophthalmologica.

[22] Baker H, Cousens SN, Murdoch IE (2010). Poor public health knowledge about glaucoma: fact or fiction?. Eye (Lond).

[23] Wu PX, Guo WY, Xia HO, Lu HJ, Xi SX (2011). Patients’ experience of living with glaucoma: a phenomenological study. J Adv Nurs.

[24] Lewallen S, Hassan HG, Al Attas AH, Courtright P (2011). A population-based study of care-seeking behavior in rural Tanzanians with glaucoma blindness. J Glaucoma.

[25] Gilmour-White JA, Shah P, Cross V, Makupa W, Philippin H (2015). Glaucoma awareness and access to healthcare: perceptions among glaucoma patients in Tanzania. Postgrad Med J.

[26] Global health and foreign policy (2012). A/67/L.36. United Nations. http://www.un.org/ga/search/view_doc.asp?Symbol=A/67/L.36&referer=http://www.un.org/en/ga/info/draft/index.shtml&Lang=E.

[27] WHO (2013). Universal eye health: a global action plan for the prevention of avoidable blindness and visual impairment 2014–2019. http://www.who.int/entity/blindness/EyeHealthActionPlanWHA66.pdf?ua=1.

[28] World Health Organization (2007). Vision2020 The Right to Sight Global initiative for the elimination of avoidable blindness Action Plan 2006–2011. http://www.who.int/blindness/Vision2020_report.pdf.

[29] Farmer PE, Nizeye B, Stulac S, Keshavjee S (2006). Structural violence and clinical medicine. PLoS Med.

[30] Galtung J (1969). Violence, peace and peace research. J Peace Res.

[31] Farmer P, Pathologies of power (2005). Health, human rights, and the new war on the poor.

[32] Taylor JS Explaining difference: ‘Culture’, ‘Structural Violence’, and Medical Anthropology. http://www.washington.edu/omad/ctcenter/projects-common-book/mountains-beyond-mountains/explaining-difference/.

[33] Tunde-Ayinmode MF, Akande TM, Ademola-Popoola DS (2011). Psychological and social adjustment to blindness: understanding from two groups of blind people in Ilorin, Nigeria. Ann Afr Med.

[34] Glen FC, Crabb DP (2015). Living with glaucoma: a qualitative study of functional implications and patients’ coping behaviours. BMC Ophthalmol.

[35] Last M, Prince RJ, Marsland R (2013). The peculiarly political problem behind Nigeria’s primary health care provision. Making and unmaking public health in Africa. Ethnographic and historical perspectives.

[36] Last M (1981). The importance of knowing about not knowing. Soc Sci Med B.

[37] Gilbert CE, Murthy GV, Sivasubramaniam S, Kyari F, Imam A, Rabiu MM (2010). Couching in Nigeria: prevalence, risk factors and visual acuity outcomes. Ophthalmic Epidemiol.

